# Human astrovirus infection associated with encephalitis in an immunocompetent child: a case report

**DOI:** 10.1186/s13256-019-2302-6

**Published:** 2019-11-23

**Authors:** Georgia Koukou, Sandra Niendorf, Britt Hornei, Jan-U Schlump, Andreas C. Jenke, Sonja Jacobsen

**Affiliations:** 1 EKO Children’s Hospital, Witten/Herdecke University, Virchowstr. 20, Oberhausen, Germany; 2 Consultant Laboratory for Noroviruses, Unit Viral Gastroenteritis and Hepatitis Pathogens and Enteroviruses, Department of Infectious Diseases, Robert Koch Institute, Seestraße 10, 13353 Berlin, Germany; 3 EKO Department of Laboratory Medicine and Microbiology, Virchowstr. 20, Oberhausen, Germany; 4 Children’s Hospital Kassel, Witten/Herdecke University, Alfred-Herrhausen Str. 40, Witten, Germany

**Keywords:** Classic human astroviruses, CNS infections, Encephalitis, Immunocompetent, Gastroenteritis

## Abstract

**Background:**

Until today, classic human astroviruses have not been associated with central nervous system infections in immunocompetent patients.

**Case presentation:**

A 16-month-old Caucasian girl presented with repetitive generalized seizures with a 4-day history of watery diarrhea, which had already gradually improved. Initially, the prolonged seizures ceased after systemic midazolam treatment and were thought to be fever associated. However, her mental status remained altered, and after seizure recurrence, she was transferred to our pediatric intensive care unit. Seizure control was achieved by a combination of high-dose levetiracetam and phenobarbital, but she remained unconscious. An electroencephalogram at this time revealed generalized high voltage theta activity. All laboratory analyses, including extended blood and cerebrospinal fluid analyses, and a brain magnetic resonance imaging were normal.

On day 4, the child gradually became conscious, but was very agitated and not able to walk. Since an electroencephalogram at this time still revealed generalized high voltage theta activity, although she had not received sedative medications for 72 hours, she was diagnosed as having encephalopathy. At that time, results of diagnostic testing of the stool sample were positive for classic astrovirus infection, and we decided to analyze the initially obtained cerebrospinal fluid for astrovirus as well. Cerebrospinal fluid was also found positive for human astrovirus. Sequencing analysis revealed a classic astrovirus genotype 1 with exactly the same nucleotide sequence as in the feces. Clinically, the child gradually improved and was discharged on day 9.

**Conclusions:**

Whereas the new human astrovirus subtypes have been recently associated with central nervous system infection, this is the first case of encephalitis in an immunocompetent child due to classic human astrovirus. Considering that classic human astroviruses are the third most common etiological agents of viral gastroenteritis in children, we believe that human astroviruses as causative agents for central nervous system infections should be considered more often, especially in children and infants with preceding gastroenteritis.

## Background

Human astroviruses (HAstVs) were first identified in 1975 in stool samples from children with diarrhea. Until recently, the gastrointestinal tract had been considered the main site of infection with particularly severe acute gastroenteritis in children. Recently, highly divergent strains of astroviruses named Melbourne (MLB) and Virginia/Human-Mink-Ovine-like (VA/HMO) were discovered, which are phylogenetically markedly distant from the classic HAstV. These novel HAstVs have been identified as the cause of central nervous infections such as meningitis and encephalitis, especially in immunocompromised children with gastrointestinal symptoms. To date, only one case has been described in which the classic HAstV genotype four has been associated with central nervous system (CNS) infections in a hospitalized infant with severe combined immunodeficiency (SCID) in Switzerland [1].

## Case presentation

A 16-month-old Caucasian girl presented with prolonged, generalized, repetitive tonic-clonic seizures to our emergency department. She had watery diarrhea with a low-grade fever up to 38.4 °C and new types of seizures 4 days before. Two months earlier, she was admitted to our neuropediatric ward due to suspected generalized seizures. At that time, her psychomotor development and all clinical and laboratory findings, such as cerebrospinal fluid (CSF) status, electroencephalography as well as a cerebral magnetic resonance imaging (MRI) were normal. After a video documentation, a diagnosis of breath-holding spells was made. On admission day, she was unconscious with a generalized tonic-clonic seizure for approximately 20 minutes. At our emergency department, she received 5 mg midazolam buccal and the seizures subsided. She was then transferred to the general pediatric ward under the impression of a febrile seizure. However, during the following hours seizures reoccurred. She received levetiracetam intravenously up to a total dose of 50 mg/kg per day without a lasting effect. Full blood count, electrolytes, ammonia, blood gas analysis, inflammatory markers, and transaminases as well as ophthalmologic assessment were all repeatedly normal (Table 1). On day 2, she still had short generalized seizures and her consciousness deteriorated steadily so that a lumbar puncture was performed. Seizure control was only achieved after the application of phenobarbital (30 mg/kg) and she was then transferred to the pediatric intensive care unit. At that point, electroencephalography showed an encephalopathic picture with a generalized high amplitude theta and delta activity. Seizure activity was not noted. A CSF examination showed no abnormalities. Her glucose level was 60 mg/dl (reference range 40–70 mg/dl), protein level 12.6 mg/dl (reference range 15–45 mg/dl), lactate level 12.4 mg/dl (reference range < 20 mg/dl), and no cells were present. CSF was also negative for herpes simplex virus type 1 (HSV-1), herpes simplex virus type 2 (HSV-2), enteroviruses, and Epstein–Barr virus. Isoelectric focusing showed no oligoclonal bands, but a Reibergram revealed an intrathecal production of immunoglobulin (IgM) and immunoglobulin (IgG) indicating an acute inflammatory process (Table 2). On day 3, she was still somnolent, so that a cerebral MRI was performed which showed no abnormalities. Electrolytes and glucose levels were repeatedly within normal limits (Table 1). On day 4, she gradually became awake, but was very agitated and not able to walk. Electroencephalography revealed a generalized high voltage theta activity, even though she had not received any sedative medication for 72 hours. The results of the stool samples, which were obtained on the third day of her hospitalization, were negative for *Campylobacter*, *Salmonella*, *Shigella*, *Yersinia* species, norovirus, enterovirus, *Rotavirus*, and adenovirus except for HAstV (Table 3).

**Table 1:** Blood parameters

Complete blood count	Reference range	Day of admission	Day 3
Leukocytes	6.2–15 × 10^3^/μl	5.9 × 10^3^/μl	4.6 × 10^3^/μl
Erythrocytes	4.1–5.0 × 10^12^/μl	4.3 × 10^12^/μl	4.17 × 10^12^/μl
Hemoglobin	10.3–12.4 g/l	12.0 g/l	11.8 g/l
Hematocrit	26–37%	36%	33%
MCV	81–99 fl	82 fl	80 fl
MCH	23–28 pg/cell	28 pg/cell	28 pg/cell
MCHC	32–36 g/dl	33.8 g/dl	35.5 g/dl
Thrombocytes	130–450 × 10^3^/μl	267 × 10^3^/μl	255 × 10^3^/μl
Normoblasts %		0.1%	
Neutrophils %	21–67%	50%	
Lymphocytes %	20–64%	35%	
Monocytes %	5–11%	14%	
Eosinophils %	0–4%	1%	
Basophils %	0–1%	0%	
Chemistry panel	Reference range	Day of admission	Day 3
Urea	17–43 mg/dl	33 mg/dl	11 mg/dl
Creatinine	0.6–1.1 mg/dl	0.44 mg/dl	0.37 U/l
CK	< 247 U/l		75 U/l
GOT	< 82 U/l	75 U/l	49 U/l
GPT	< 30 U/l	17 U/l	15 U/l
LDH	< 300 U/l		241 U/l
CRP	< 0.5 mg/dl	< 0.2 mg/dl	< 0.2 mg/dl
Ammonia	16–53 μmol/l	34.9 μmol/l	41.6 μmol/l
Magnesium	0.77–1.03 mmol/l		0.77 mmol/l
Potassium	3.4–4.5 mmol/l	4.0 mmol/l	4.3 mmol/l
Sodium	136–146 mmol/l	143 mmol/l	141 mmol/l
Calcium	1.15–1.29 mmol/l	1.27 mmol/l	1.22 mmol/l
Glucose	70–105 mg/dl	74 mmol/l	87 mmol/l
Chloride	98–106 mmol/l	108 mmol/l	104 mmol/l
HbA1c%	< 6%	4.8%	
Cortisol	6.7–22.6 μg/dl	22.3 μg/dl	
ACTH	4.7–48.8 pg/ml	15.8 pg/ml	

*ACTH* adrenocorticotropic hormone, *CK* creatine kinase, *CRP* C-reactive protein, *GOT* glutamic oxaloacetic transaminase, *GPT* glutamate-pyruvate transaminase, *HbA1c* glycated hemoglobin, *LDH* lactate dehydrogenase, *MCH* mean corpuscular hemoglobin, *MCHC* mean corpuscular hemoglobin concentration, *MCV* mean corpuscular volume

**Table 2:** Cerebrospinal fluid parameters

Cerebrospinal fluid results	
Cell count	1/3 /μl
Glucose	60 mg/dl
Protein	12.6 mg/dl
Lactate	12.4 mg/dl
Epstein–Barr virus DNA	Negative
Astrovirus PCR	Positive
Herpes virus 1 and 2	Negative
Enteroviruses	Negative
Isoelectric focusing	No sign of oligoclonal bands (serum and CSF)
Reibergram	Acute inflammatory process with intrathecally produced IgG and IgM, normal CSF barrier function

*CSF* cerebrospinal fluid, *IgG* immunoglobulin G, *IgM* immunoglobulin M, *PCR* polymerase chain reaction

**Table 3:** Stool parameters

Stool results	
*Campylobacter* antigen (ELISA)	Negative
Astrovirus antigen (ELISA)	Positive
Adenovirus Antigen (ELISA)	Negative
Norovirus (PCR)	Negative
*Rotavirus* (PCR)	Negative
*Salmonella* (stool culture)	Negative
*Shigella* (stool culture)	Negative
*Yersinia* species (stool culture)	Negative
*Clostridium difficile* PCR (toxin A + B)	Negative

*ELISA* enzyme-linked immunosorbent assay, *PCR* polymerase chain reaction

**Fig. 1:**
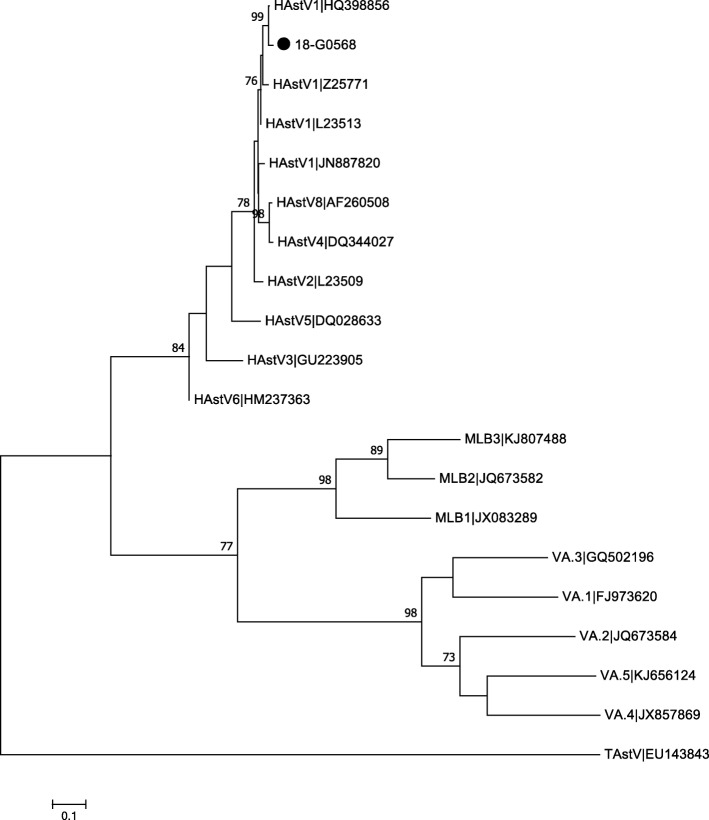
The phylogenetic tree of a 410 bp alignment of the ORF1b region of human astrovirus identified from the patient (18-G0568) belongs to the classic human astrovirus genotype human astrovirus-1. The tree was constructed using the Maximum Likelihood method and the Tamura-3-parameter method with the Bootstrap test (1000 replicates). For the modeling of the evolutionary rate, a discrete gamma distribution model was used with MEGA 7. Bootstrap values above 70 are shown. Reference sequences obtained from the GenBank are indicated by the accession number, TAstV was used as an outgroup (GenBank EU143843). *HAstV* human astrovirus, *MLB* Melbourne, *TAstV* turkey astrovirus, *VA* Virginia

We then hypothesized an astrovirus-related CNS infection and tested the initially obtained CSF sample for the virus. Sanger sequencing revealed a classic genotype HAstV-1 with the same nucleotide sequence in both samples (Fig. 1). Initially, we considered a therapy with intravenous immunoglobulin (IVIG) ± methylprednisolone, but abstained from it due to her fast and good clinical improvement [2]. Over the following days, she improved gradually, the electroencephalography normalized and on day 9 she was discharged. On follow-up over the next 6 months she showed a normal sensorimotor development without any signs of neurological impairment. Despite extensive investigations, we could not identify the source of the infection in this case. Most commonly described reservoirs and sources of infection for humans include fruits, vegetables, and water [2].

## Discussion and conclusions

HAstVs constitute the third most common viral agent of acute diarrhea after *Rotavirus* and norovirus and are responsible for up to 10% of non-bacterial gastroenteritis [3]. However, the recently discovered highly divergent HAstVs, named MLB and VA/HMO, have been identified as the cause of CNS infections in vulnerable individuals highlighting that these viruses bypass the gastrointestinal tract and infect other tissues and organs [4]. HAstV-VA1/HMO-C has also been detected in the brain tissue of immunocompromised patients with acute encephalitis. Overall, CNS infection with the newly described HAstVs almost exclusively occurs in immunocompromised patients [5].

Here, we report the first case of a CNS infection with a classic HAstV in a non-immunocompromised infant. However, common CSF findings of CNS infections such as pleocytosis were in our case completely normal, which is in line with previous reports [1]. Diagnosis of an astrovirus CNS infection is further complicated by the unreliable detectability of acute IgM antibodies in CSF and the fact that even serum antibodies in the acute phase of infection were detectable only in a few patients [6]. Therefore, the diagnosis of an acute astrovirus CNS infection currently relies exclusively on the clinical picture, specific detection of astrovirus in the CSF mostly by next-generation sequencing (NGS)-based diagnosis, and exclusion of other known causes for encephalitis, as in this case.

Similar to previous reports on immunocompromised patients and infection of HAstV-MLB or VA strains, the most probable route of infection was enteric inoculation followed by viral translocation and infection of the CNS via the bloodstream. In previous case reports, HAstV-VA1 was found in neurons and astrocytes [7]. A recent publication showed for the first time that the novel described HAstV-VA1 could be propagated in cell culture without trypsin, which was mandatory for the cell culture of classic HAstV strains. The authors supposed that classic and novel HAstV genotypes react differently toward trypsin for the proteolytic cleavage of the viral capsid. This may influence the contribution of the tissue tropism of HAstVs and novel HAstV strains might spread toward tissues with low trypsin levels; however, so far the mechanism of neuroinvasion of HAstV remains unclear [8]. Interestingly, CNS infections in mammals such as swine, sheep, and cattle seem to be quite common [9].

We therefore believe that in the future the possibility of HAstV CNS infection needs to be considered on a routine basis in non-immunocompromised infants and young children with sensitive detection systems covering all HAstV species (classic HAstV genotypes 1 to 8 as well as HAstV-MLB and VA strains). Even though there are currently no specific therapeutic options available, the identification of the causative agent in an encephalopathic child is of utmost importance – for the community to implement prophylactic actions and even more for the parents who always seek to obtain an explanation – in particular, in possibly very debilitating diseases such as encephalopathy/encephalitis.

## Data Availability

All data generated or analyzed during this study are included in this published article. The datasets used and/or analyzed during the current study are available from the corresponding author on reasonable request.

## Abbreviations

CNS: Central nervous system
CSF: Cerebrospinal fluid
HAstV: Human astrovirus
HSV-1: Herpes simplex virus type 1
HSV-2: Herpes simplex virus type 2
IgG: Immunoglobulin G
IgM: Immunoglobulin M
IVIG: Intravenous immunoglobulin
MLB: Melbourne
MRI: Magnetic resonance imaging
NGS: Next-generation sequencing
SCID: Severe combined immunodeficiency
VA/HMO: Virginia/Human-Mink-Ovine-like
